# Neuroprotective effects of Simvastatin against alcohol-induced oxidative stress and neurodegeneration in the Hippocampus of adolescent mice

**DOI:** 10.1007/s11011-025-01668-w

**Published:** 2025-07-09

**Authors:** Robin du Preez, Tabo Mwila, Alice Efuntayo, Oladiran I. Olateju

**Affiliations:** Department of Anatomical Sciences, School of Biomedical Sciences, Faculty of Health Sciences, University of the Witwatersrand, 7 York Road, Parktown, Johannesburg, 2193 Republic of South Africa

**Keywords:** Alcohol, Adolescent, Simvastatin, Oxidative stress, Neuroprotection, Hippocampus, Neurogenesis

## Abstract

Adolescent alcohol abuse in disadvantaged communities is a significant concern due to regulatory gaps. It disrupts brain development, particularly affecting the hippocampus, which is vulnerable to alcohol-induced oxidative stress, resulting in impaired neuronal signalling, increased cell death, and reduced neurogenesis. Simvastatin, a cholesterol-lowering drug, has neuroprotective and antioxidant effects, but its potential in protecting against alcohol-related brain damage is unclear. This study examined the protective effects of Simvastatin in four-week-old C57BL/6J mice administered 20% alcohol (intraperitoneal, i.p.), 5 or 15 mg/kg Simvastatin orally, followed by 20% alcohol (i.p.) or the controls (i.e., 5 mg/kg Simvastatin only or no treatment). After 28 days, the harvested brains underwent biochemical or immunohistochemical (IHC) analysis. Biochemical analyses measured malondialdehyde (MDA) levels, glutathione peroxidase (GSH-Px) and superoxide dismutase (SOD) activity in homogenised hippocampal samples and IHC involved immunolabelling for PcNA or DCX. PcNA- or DCX-positive cells in the suprapyramidal blade of the dentate gyrus were counted using QuPath software. Alcohol elevated GSH-Px activity, indicating oxidative damage, but both Simvastatin concentrations reduced this, with 15 mg being more effective in females. MDA level and SOD activity remained unchanged. Simvastatin at 5 mg reduced alcohol’s effect on PcNA-positive cells in both sexes, while 15 mg was more effective in females. For DCX-positive cells, 5 mg Simvastatin was protective in both sexes, but 15 mg showed no effect. Overall, Simvastatin exhibited antioxidant and neuroprotective effects against alcohol-induced hippocampal damage, suggesting its potential for treating alcohol-related brain disorders.

## Introduction

Ethanol, commonly known as alcohol, is the oldest and most widely used recreational substance globally (Marshall 2014; Dey and Meenu Singh 2015; Jones 2022). Its consumption is deeply ingrained in society, cutting across all socio-economic backgrounds. However, its widespread availability contributes to patterns of misuse, particularly among adolescents (Reddy et al. 2013; Morojele and Ramsoomar 2016; Letsela et al. 2019). Adolescence, generally ranging from ages 10 to 19, is a crucial developmental period characterised by behavioural, hormonal, and neurochemical changes that equip individuals for independent adulthood (Carvajal and Lerma-Cabrera 2015). This phase is often accompanied by increased exploration and risk-taking, including experimentation with alcohol (Steinberg and Morris 2001; Carvajal and Lerma-Cabrera 2015). While adolescents may drink less frequently than adults, they tend to consume larger quantities per occasion, leading to a higher prevalence of binge drinking (MacPherson et al. 2010; Chung et al. 2018). As a result, adolescents face an increased risk of alcohol-related illnesses and neurocognitive impairments (Hermens and Lagopoulos 2018). These health consequences not only affect individual well-being but also place a significant financial burden on government resources (Harrison et al. 2010; Scott-Sheldon et al. 2013; Khuzwayo et al. 2020).

The generation of new neurons in the hippocampal dentate gyrus (DG) is influenced by an interplay of both internal (e.g., growth factors) and external (e.g., environmental conditions, drugs) factors (Åberg et al. 2005; Cameron and Glover 2015; Opendak et al. 2016). The flow of information within the hippocampal circuitry (White et al. 2000; White and Swartzwelder 2004) is hindered by alcohol, making the hippocampus highly susceptible to the neurotoxic effects of alcohol, as this region continues to undergo refinement and maturation throughout adolescence into early adulthood and plays a key role in the integration of emotion, reward processing, homeostasis, and memory (Kutlu and Gould 2016; Mira et al. 2019; Walker et al. 2021).

Given the damaging effects of alcohol on hippocampal function, it is crucial to explore interventions that can mitigate or prevent alcohol-related brain damage. One promising candidate is Simvastatin, a widely used cholesterol-lowering drug with pleiotropic effects and potential for drug repurposing (Ardakani et al. 2023). Simvastatin, similar to other statins, functions by blocking HMG-CoA reductase, the key enzyme in the mevalonate (MVA) pathway, which leads to a reduction in cholesterol production in hepatocytes (Holstein and Hohl 2004; Schachter 2005). Beyond its cardiovascular benefits, Simvastatin exhibits anti-inflammatory, antioxidant, and immunomodulatory properties, improves endothelial function, and stabilises atherosclerotic plaques (Blum and Shamburek 2009; Palaniswamy et al. 2010; Kadhim et al. 2020; Liu et al. 2023). These neuroprotective properties make it a strong candidate for combating alcohol-induced brain damage.

Statins support neurological recovery by reducing oxidative stress, enhancing vascular function, and modulating inflammatory responses through microglial activation (Stepień et al. 2005; Tapia-Perez et al. 2007). Research suggests statins aid recovery from spinal cord injuries, stroke, intracerebral haemorrhage, and epileptic seizures (Asahi et al. 2005; Gomis et al. 2010; Nazli et al. 2015; Vitturi and Gagliardi 2020). In addition, statins may contribute to the prevention of neurodegenerative diseases, including Alzheimer’s, Parkinson’s, Huntington’s, and Multiple Sclerosis, and contribute to the prevention of mental health disorders like depression and anxiety (Tramontina et al. 2011; Kim et al. 2014; Ahmed et al. 2016; Carroll et al. 2019). Given these potential benefits, further investigation is warranted into the effectiveness of Simvastatin against alcohol-related neurodegeneration.

Statins are among the most widely prescribed medications globally, owing to their potent effects on lipid regulation and pleiotropic properties (McFarland et al. 2014; Bedi et al. 2016; Kosowski et al. 2021). They are also well-tolerated and remarkably safe (Schachter 2005; Hu et al. 2012; Maji et al. 2013). While the neuroprotective potential of statins has been widely explored (Whyte et al. 2019; Kosowski et al. 2021; Nasef et al. 2021), their effectiveness against alcohol-induced brain damage remains less understood. This study, therefore, aimed to assess the neuroprotective and antioxidant properties of Simvastatin in the hippocampus of adolescent mice exposed to alcohol. The findings could offer valuable insights into the potential of Simvastatin in mitigating alcohol-induced brain damage, which could aid in the management of alcohol-related brain disorders.

## Materials and methods

### Animal study and experimental design

This study was approved by the Animal Research Ethics Committee (AREC) at the University of the Witwatersrand, Johannesburg, South Africa (Ethics Clearance Number: 2019/11/63/C). C57BL/6J mice aged between 4 and 8 weeks (defined as adolescence according to Lach et al. (2020) were sourced from the National Health Laboratory Services (NHLS), Johannesburg. Both male and female mice were included to account for potential sex-specific differences in drug response. Mice of the same sex were group-housed in polycarbonate cages (200 × 200 × 300 mm) at the Wits Research Animal Facility (WRAF), Faculty of Health Sciences, University of the Witwatersrand. The animal holding room operated on a reversed 12-hour light/dark cycle, with lights off from 06:00 to 18:00. Environmental enrichment was provided in accordance to the WRAF’s Standard Operating Procedures (SOP) for mouse care.

In line with methodologies described by Nchodu et al. (2024a, b), three-week-old C57BL/6J mice of both sexes (*n* = 5 males and 5 females per group) were randomly allocated to one of five experimental groups: Control (C) – no treatment; Alcohol-only group (ALC) – 2.5 g/Kg/day of 20% alcohol administered via intraperitoneal (i.p.) injection; Simvastatin-only group (SIM) – 5 mg/Kg/day Simvastatin administered via oral gavage; Alcohol + Simvastatin 5 mg (ALC + SIM5) – 5 mg/Kg/day of Simvastatin via oral gavage followed by 2.5 g/Kg/day of 20% alcohol via i.p. injection; and Alcohol + Simvastatin 15 mg (ALC + SIM15)– 15 mg/Kg/day Simvastatin via oral gavage followed by 2.5 g/Kg/day of 20% alcohol via i.p. injection. All mice were acclimatised for one week prior to the initiation of treatments. Simvastatin (Cat. No: 1612700, Merck, South Africa) was prepared fresh daily according to the protocol outlined by McKay et al. (2004). Alcohol (pharmacological-grade absolute ethanol, 99.9%; Sigma-Aldrich, South Africa; Cat. No: 24105) was diluted in sterile 0.9% saline to achieve a 20% (v/v) solution. Both drugs were filter-sterilised prior to administration. All procedures involving oral gavage and intraperitoneal injections were performed by trained personnel at the Wits Research Animal Facility (WRAF) to ensure animal welfare. Treatments were administered once daily for 28 consecutive days. Throughout the experimental period, mice had ad libitum access to standard chow and water. However, food and water were withheld for approximately two hours in non-alcohol-treated groups to partially mimic the feeding suppression associated with alcohol exposure.

On the final day of treatment, blood samples were obtained 30 min after the intraperitoneal alcohol injection from all mice that received alcohol. This time point corresponds with peak blood alcohol concentration (BAC) as stated in previous studies (Donovan 2009; Mitchell et al. 2014). A small incision was made at the saphenous vein on the hind limb, and approximately 50 µl of blood was collected from each mouse using a heparinised capillary tube. Samples were stored at 4 °C overnight and then centrifuged for 15 min at 5000 rpm using Vivaspin500™ 100 μm membrane tubes (Biotech, South Africa) to isolate serum. BAC was determined from the resulting serum using the EnzyChrom™ Ethanol Assay Kit (Cat no: MAK076-1KT, BioVision, South Africa). The average BAC values across the alcohol-treated groups ranged from 178.85 to 384.36 mg/dL.

### Tissue processing and biochemical analyses

Upon completion of the treatment period (PND 56), mice intended for biochemical analyses were euthanised by decapitation, in line with the WRAF’s SOP. Immediately post-mortem, the brains were quickly removed from the skulls, weighed, and recorded. Each brain was then divided into left and right cerebral hemispheres in ice-cold 0.1 M phosphate-buffered (PB) solution under a dissecting microscope. From the left hemisphere, the prefrontal cortex, cerebellum, midbrain, and thalamus were carefully removed to expose the hippocampus. The hippocampus was then promptly excised, snap-frozen in liquid nitrogen and stored at − 80 °C for subsequent biochemical analyses, including quantification of malondialdehyde (MDA) levels, glutathione peroxidase (GSH-Px), and superoxide dismutase (SOD) enzymatic activities.

Samples were homogenised in 10 times their volume (weight-to-volume ratio) of 0.01 M PBS (pH 7.4). The homogenates were centrifuged in Eppendorf tubes at 10,000 rpm for 10 min. The clear supernatant was carefully collected, and the protein concentration in each sample was quantified using the Pierce™ BCA Protein Colorimetric Assay Kit (Cat no: 23227, ThermoFisher Scientific), with bovine serum albumin (BSA) as the reference standard. All procedures followed the manufacturer’s guidelines. The protein concentrations were measured on a microplate reader at 562 nm (A51119500C, Multiskan SkyHigh Microplate Spectrophotometer, ThermoFisher Scientific) and used for further biochemical analyses.

### Malondialdehyde (MDA) levels

Lipids are essential structural components of neuronal membranes and bioactive signalling molecules in neurons (Tracey et al. 2021). However, they are highly susceptible to oxidative stress, with polyunsaturated fatty acids being particularly vulnerable to oxidative damage (Lushchak and Bagnyukova 2006; Monaghan et al. 2009). In this study, MDA levels were assessed as a marker of oxidative stress in the hippocampus. MDA was quantified using a colorimetric assay kit (Cat no: E-BC-K025-M; Elabscience) following the manufacturer’s guidelines, with absorbance measured at 532 nm. Aliquots of the homogenate from the protein extraction were used in all the assays. MDA levels were calculated based on a standard curve, with results expressed as micromoles per gram of protein (µmol/g protein).

### Glutathione peroxidase (GSH-Px) activity

GSH-Px activity was measured using a commercial assay kit (Cat. no: E-BC-K096-M; Elabscience^®^) following the manufacturer’s instructions, with absorbance readings taken at 412 nm. The assay relies on the reduction of hydrogen peroxide (H_2_O_2_) by GSH-Px, during which reduced glutathione (GSH) is converted to oxidized glutathione (GSSG). GSH-Px activity was quantified using a standard curve, and results were expressed as units per milligram of protein (U/mg protein).

### Superoxide dismutase (SOD) activity

SOD catalyses the conversion of superoxide anion into H_2_O_2_ and molecular oxygen, playing a vital role in antioxidation. SOD activity was determined using the SOD Determination Kit (Cat no: 19160, Sigma-Aldrich), following the manufacturer’s guidelines. The assay uses a water-soluble tetrazolium salt that, when reduced by superoxide anions (O_2_^−^), forms a water-soluble formazan dye. The reduction rate, which is directly linked to xanthine oxidase activity, is inhibited by SOD. Absorbance at 440 nm, inversely related to the superoxide anion concentration, was measured to assess SOD activity. Results were calculated using the provided equation and expressed as units per millilitre (U/ml).

### Tissue processing and immunohistochemistry

At the end of the treatment period (PND 56), mice designated for immunohistochemical analysis were euthanised using Euthanaze (sodium pentobarbital, 80 mg/kg, i.p.). The animals were transcardially perfused with saline, followed by 4% paraformaldehyde (PFA) in 0.1 M phosphate buffer (PB). The brains were extracted, weighed, and post-fixed in 4% PFA at 4 °C for 24 h. The brain samples were then cryo-protected in a 30% buffered sucrose solution in 0.1 M PB.

The left brain hemispheres were sectioned at 50 μm in the sagittal plane using a freezing-stage microtome. One to three serial sections were collected and placed in a 24-well plate with 0.01 M PB. Endogenous peroxidase activity was blocked by immersing the sections in an inhibitor solution (49.2% methanol, 49.2% 0.1 M PB, 1.6% 30% H_2_O_2_) for 30 min at room temperature, followed by three 10-minute washes in 0.01 M PB. Unspecific binding was blocked by pre-incubating the sections in a blocking buffer (0.25% Triton X-100 in 0.1 M PB, 3% normal goat serum, 2% BSA) for 2 h at room temperature. Every third section was immunolabelled with either anti-proliferating cell nuclear antigen (PcNA) (1:200 mouse anti-PcNA; Cat no: MAB424R, Merck) to label proliferating cells in the G1/S phase, or anti-doublecortin (DCX) (1:2000 rabbit anti-DCX; Cat no: ab18723, Abcam) to label immature neurons. The sections were incubated for 48 h at 4 °C under gentle agitation.

The sections were subsequently incubated in corresponding secondary antibodies: for anti-PcNA, a 1:1000 goat anti-mouse IgG biotinylated (Cat no: BA-9200, Vector Labs), and for anti-DCX, a 1:1000 goat anti-rabbit IgG biotinylated (Cat no: BA-1000, Vector Labs) for 2 h at room temperature. Sections were then incubated in an avidin-biotin solution (1:125; Vectastain^®^ Elite^®^ ABC-HRP Kit, Peroxidase; Cat no: PK-6100) for 1 h. For chromogen development, the sections were immersed in a 0.05% diaminobenzidine (DAB) solution in 0.1 M PB for 15 min, followed by the addition of 3.3 µl of 30% hydrogen peroxide/1 ml of DAB solution. The DAB reaction was stopped by adding 2 mL of 0.1 M PB at room temperature. The sections were then mounted on 0.5% gelatin-coated glass slides, dried, and stained for Nissl to visualise general brain morphology. There were no observable morphological differences across the groups for both sexes (results not shown). The processed sections were dehydrated in an alcohol series, cleared in xylene, and coverslipped with a DPX mounting medium.

### Quantification of PcNA- and DCX-positive cell populations in the dentate gyrus

The quantity of PcNA- and DCX-positive cells in the left suprapyramidal blade of the DG was measured across experimental groups and both sexes. To evaluate the distribution of stained cells, tissue sections were scanned using a VS200 Slide Scanner (Evident, USA). The scanned images were subsequently analysed using QuPath software (Version 0.5.0; USA). PcNA- or DCX-positive cells were counted along the full rostro-caudal axis of the suprapyramidal blade using QuPath’s counting point annotation tool. Only cells located within or touching the subgranular zone (SGZ) were included in the count. The length (in µm) of the suprapyramidal blade was determined using the polyline annotation tool in QuPath. The cell distribution per suprapyramidal blade was calculated by dividing the total number of PcNA- or DCX-positive cells by the measured length of the corresponding suprapyramidal blade, following the method outlined by Olateju et al. 2021.

### Statistical analyses

Descriptive statistics (mean ± SD or median) were performed. Statistical analyses were conducted using Paleontological Statistics (PAST) software (version 4.03). The Shapiro-Wilk test was applied to assess data normality, followed by either a One-Way ANOVA or Kruskal-Wallis test to compare the means or medians of the measurements (brain/body mass index, MDA level, GSH-Px and SOD activities, distribution of PcNA- or DCX-positive cells) across groups. Post-hoc analyses were conducted using Tukey’s or Dunn’s test to identify significant differences between groups. Boxplots were created using Excel (Microsoft Office Pro, USA). A p-value of less than 0.05 was deemed statistically significant.

## Results

### General observations on the brain and body mass across experimental groups

The average body and brain masses, as well as the brain/body ratio, are shown in Table 1. In male mice, no statistically significant differences were observed in either body mass, brain mass, or brain/body mass ratio across all treatment groups (*p* > 0.05), indicating that the treatments had no major impact on these parameters in male mice. In contrast, significant differences were observed in the female mice. On Day 1, body mass differed significantly among treatment groups, with post hoc tests revealing differences between the following: SIM < ALC (*p =* 0.039), ALC + SIM5 < ALC (*p =* 0.004), and ALC + SIM5 < ALC + SIM15 (*p =* 0.037). By Day 28, although body mass (*p =* 0.069) and brain mass (*p =* 0.453) showed no significant changes, the brain/body mass ratio was significantly different (*p =* 0.023). Specifically, the SIM group had a higher brain/body ratio compared to the control (*p =* 0.035) or ALC (*p =* 0.001). The ALC group showed a lower ratio compared to ALC + SIM5 (*p =* 0.043) or ALC + SIM15 (*p =* 0.037), while the brain/body mass ratio was similar in the SIM, ALC + SIM5, and ALC + SIM15 groups.

**Table 1:** Summary of the body mass, brain mass, and brain/Body mass ratio of mice across different experimental groups and both sexes

		Control	SIM	ALC	ALC + SIM5	ALC + SIM15	*p*
Female							
Day 1	Body mass (mean ± SD) (g)	12.7 ± 0.57	12.4 ± 0.22^a^	13.2 ± 0.57^a, b^	12.1 ± 0.55^b, c^	13.0 ± 0.87^c^	**0.042**
Day 28	Brain mass (mean ± SD) (g)	0.42 ± 0.01	0.42 ± 0.02	0.41 ± 0.01	0.40 ± 0.02	0.40 ± 0.02	0.453
	Body mass (mean ± SD) (g)	15.4 ± 0.65	14.3 ± 1.04	15.7 ± 0.84	14.2 ± 1.04	14.2 ± 0.98	0.069
	Brain/Body ratio (mean ± SD)	0.027 ± 0.002^a^	0.030 ± 0.002^a, b^	0.026 ± 0.002^b, c,d^	0.028 ± 0.001^c^	0.028 ± 0.001^d^	**0.023**
Male							
Day 1	Body mass (mean ± SD) (g)	15.2 ± 0.76	14.5 ± 0.35	14.5 ± 2.06	14.2 ± 0.57	14.1 ± 2.01	0.739
Day 28	Brain mass (mean ± SD) (g)	0.42 ± 0.04	0.43 ± 0.01	0.41 ± 0.02	0.42 ± 0.01	0.40 ± 0.01	0.145
	Body mass (mean ± SD) (g)	18.3 ± 1.48	18.3 ± 1.64	16.8 ± 1.35	17.2 ± 1.44	16.2 ± 2.95	0.329
	Brain/Body ratio (mean ± SD)	0.023 ± 0.001	0.023 ± 0.002	0.025 ± 0.002	0.024 ± 0.002	0.025 ± 0.005	0.69

SD = Standard deviation

^a, b,c, d^ The same letter indicates paired groups that are statistically significantly different at *p* < 0.05

### Effects on oxidative stress markers and antioxidant enzyme activities

#### Malondialdehyde (MDA) levels

**Fig. 1:**
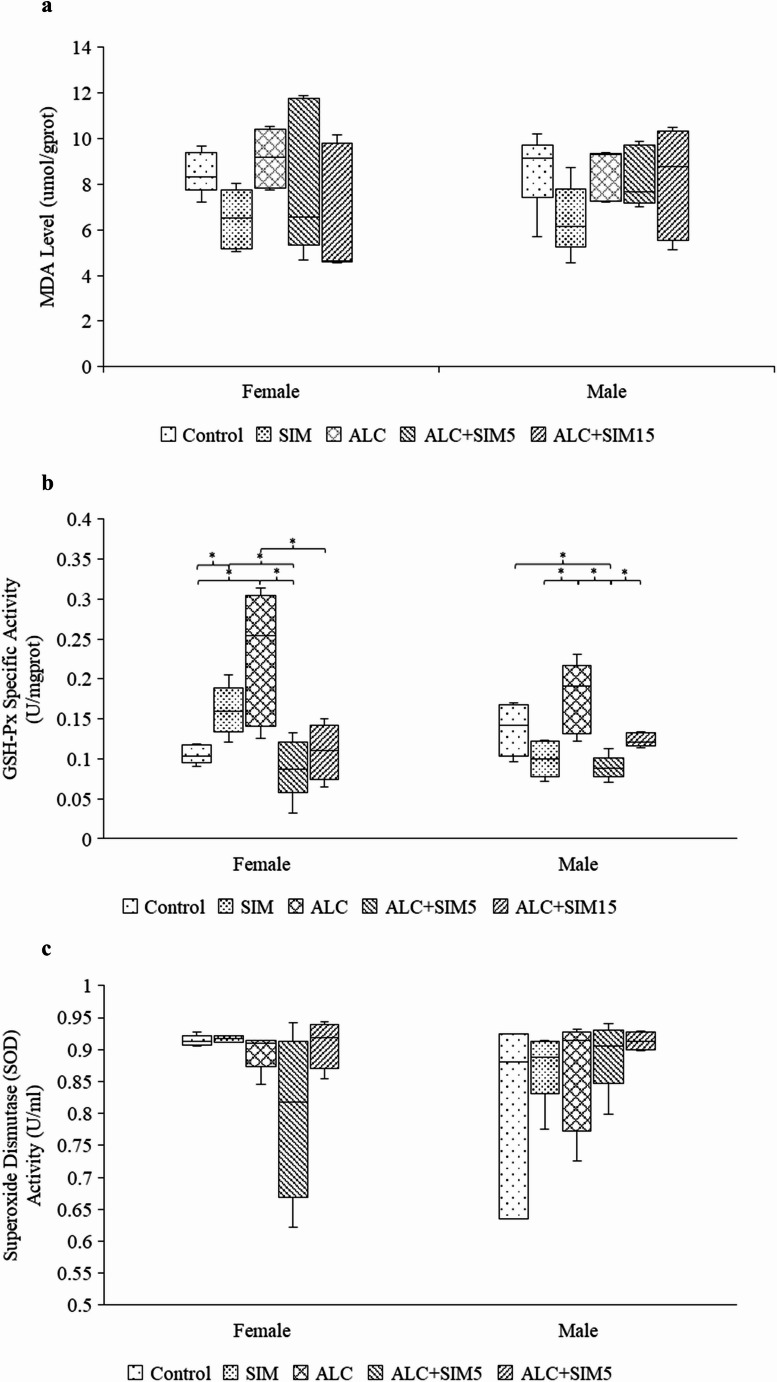
Box plots presenting the statistical comparisons of (**a**) malondialdehyde (MDA) levels (µmol/g protein), (**b**) glutathione peroxidase (GSH-Px) activity (U/mg protein), and (**c**) superoxide dismutase (SOD) activity (U/mL) in the hippocampi of female and male adolescent mice across the experimental groups. No significant differences were observed in MDA levels or SOD activities in both sexes. Both sexes showed elevated GSH-Px activity in the ALC group relative to the other groups, suggesting increased oxidative stress induced by alcohol exposure. Notably, Simvastatin treatment reduced the alcohol-induced effects in both sexes. Control – No treatment; SIM – 5 mg/kg Simvastatin; ALC – Alcohol; ALC + SIM5 – Alcohol + 5 mg/kg Simvastatin; ALC + SIM15 – Alcohol + 15 mg/kg Simvastatin. Females = 25; Males = 25. *Statistically significant at *p* < 0.05

In both the female and the male (Fig. 1a) mice, no significant differences in MDA levels were observed across the experimental groups (female: *p =* 0.231; male: *p* = 0.245).

#### Glutathione peroxidase (GSH-Px) activity

In female mice (Fig. 1b), GSH-Px activity was significantly different across experimental groups (*p =* 0.004). Specifically, the ALC group showed significantly higher GSH-Px activity compared to the control (*p =* 0.007), ALC + SIM5 (*p =* 0.002), or ALC + SIM15 (*p =* 0.021) groups, indicating an increase in enzymatic antioxidant activity in response to alcohol-induced oxidative stress. Additionally, GSH-Px activity in the control was significantly lower than in the SIM group (*p =* 0.032), and ALC + SIM5 had similar activity to the control, compared to SIM (*p =* 0.011).

In male mice (Fig. 1b), GSH-Px activity also varied significantly across experimental groups (*p =* 0.005). The ALC group exhibited significantly higher GSH-Px activity compared to the SIM (*p =* 0.006) and ALC + SIM5 (*p =* 0.001) groups, suggesting enhanced antioxidant activity in response to alcohol exposure. In contrast, GSH-Px activity in the control group was similar to that in the ALC + SIM5 group (*p =* 0.020), and ALC + SIM15 showed similar levels to SIM when compared to ALC + SIM5 (*p =* 0.05). The results indicate that alcohol exposure significantly increases GSH-Px antioxidant activity, while Simvastatin, especially at the lower dose (5 mg), modulates these effects.

#### Superoxide dismutase (SOD) activity

SOD activity is typically expressed as the amount of enzyme required to inhibit the reduction of a specific substrate by 50%. In both sexes, no significant differences in SOD activity were observed among the experimental groups (*p =* 0.298 for females and *p =* 0.532 for males) (Fig. 1c).

### Effects on neurogenic capacity

#### General Morphology of the Hippocampus in Histological and Immunohistological Sections

**Fig. 2:**
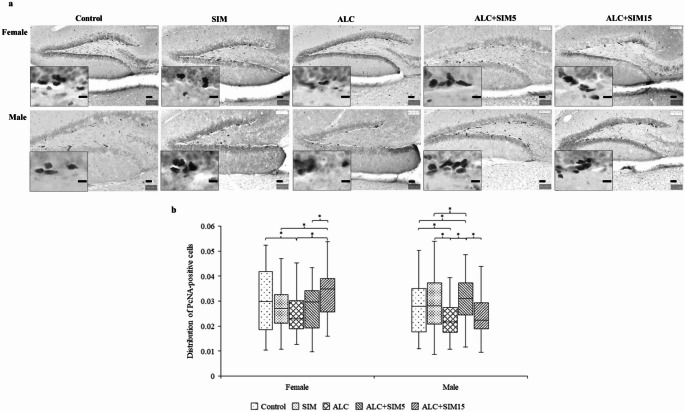
Illustrations of the (**a**) photomicrographs showing PcNA-positive cells in the dentate gyrus (**b**) and boxplots illustrating the distribution of PcNA-positive cells in the suprapyramidal blade of the dentate gyrus in female and male adolescent mice. PcNA-positive cells appeared dark and were predominantly located in the subgranular zone of the dentate gyrus, consistent with the typical morphology observed in mice. In both sexes, the ALC group showed the lowest distribution of PcNA-positive cells. Simvastatin demonstrated a neuroprotective effect against alcohol exposure, as reflected by the increased presence of PcNA-positive cells in the SIM, ALC + SIM5, and ALC + SIM15 groups, though the extent of this effect varied between sexes. Control - No treatment (No of suprapyramidal blade assessed: F = 48; M = 64); SIM – 5 mg Simvastatin (No of suprapyramidal blade assessed: F = 60; M = 70); ALC – Alcohol (No of suprapyramidal blade assessed: F = 49; M = 51); ALC + SIM5 - Alcohol + 5 mg Simvastatin (No of suprapyramidal blade assessed: F = 58; M = 60); ALC + SIM15 - Alcohol + 15 mg Simvastatin (No of suprapyramidal blade assessed: F = 42; M = 44). Female = 25; males = 25. *Statistically significant at *p* < 0.05. Scale bar of lower magnification = 50 μm; Scale bar of higher magnification = 20 μm

**Fig. 3:**
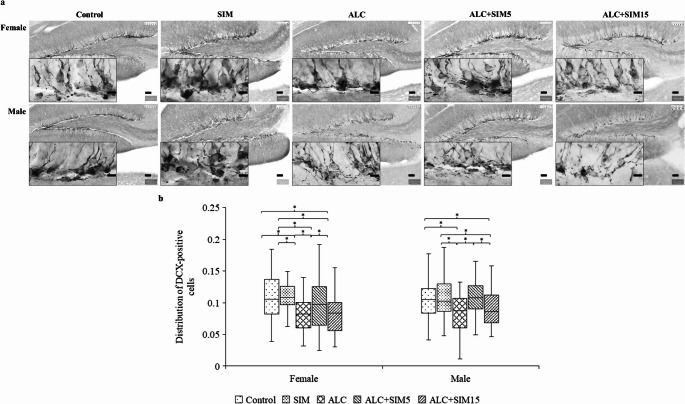
Illustrations of the (**a**) photomicrographs showing DCX-positive cells in the dentate gyrus (**b**) and the boxplots illustrating the statistical comparison of the distribution of DCX-positive cells in the suprapyramidal blades of the dentate gyrus in the hippocampus of both female and male adolescent mice. The DCX-positive cells appeared dark, and no differences in their patterns were observed across the experimental groups. In both sexes, the distribution of DCX-positive cells was lowest in the ALC group. Simvastatin demonstrated a neuroprotective effect against alcohol, as shown by the increased distribution of DCX-positive cells in the SIM and ALC + SIM5 groups. The lower concentration of Simvastatin (5 mg) was more effective in counteracting the suppressive effects of alcohol in both sexes. Control - No treatment (No of suprapyramidal blade assessed: F = 58; M = 69); SIM – 5 mg Simvastatin (No of suprapyramidal blade assessed: F = 69; M = 76); ALC – Alcohol (No of suprapyramidal blade assessed: F = 57; M = 52); ALC + SIM5 – Alcohol + 5 mg Simvastatin (No of suprapyramidal blade assessed: F = 62; M = 65); ALC + SIM15 – Alcohol + 15 mg Simvastatin (No of suprapyramidal blade assessed: F = 48; M = 48). Female = 25; Males = 25. *Statistically significant at *p* < 0.05. Scale bar of lower magnification = 50 μm; Scale bar of higher magnification = 20 μm

No observable differences in the general brain morphology (i.e., overall brain structure) were found across the treatment groups for both sexes in the PcNA- (Fig. 2a) or DCX-immunolabelled sections (Fig. 3a). Specifically, there were no differences in the DG of the hippocampus between the groups. Positively immunolabelled PcNA and DCX cells were localised within the distinct proliferative region of the SGZ of the DG. Additionally, the suprapyramidal and infrapyramidal blades of the DG were distinguishable, with well-defined boundaries across all experimental groups in the mice assessed.

#### Distribution of PcNA-positive cells

In the female mice (Fig. 2b), the distribution of PcNA-positive cells significantly differed across the experimental groups (*p =* 0.003). Specifically, the ALC group showed a significantly lower distribution of PcNA-positive cells compared to the control group (*p =* 0.015), indicating alcohol’s damaging effect on hippocampal neurogenesis. The ALC + SIM15 group had a significantly higher distribution of PcNA-positive cells compared to the ALC (*p =* 0.000), SIM (*p =* 0.006), or ALC + SIM5 groups (*p =* 0.008).

A similar trend was observed in the male mice (Fig. 2b), where the distribution of PcNA-positive cells significantly differed across the experimental groups (*p =* 0.000). The distribution of PcNA-positive cells was significantly lower in the ALC group compared to the control (*p =* 0.024), SIM (*p =* 0.002), or ALC + SIM5 (*p =* 0.000), further suggesting alcohol-induced damage to hippocampal neurogenesis. The distribution of PcNA-positive cells in the control group was similar to that in the ALC + SIM5 group (*p =* 0.017). However, the distribution of PcNA-positive cells in the ALC + SIM15 group was significantly different compared to SIM (*p =* 0.042) and ALC + SIM5 (*p =* 0.001), with levels similar to ALC. In both sexes, Simvastatin mitigated alcohol-induced damage to hippocampal neurogenesis, with the higher concentration being more effective in females and the lower concentration more effective in males. This suggests sex-specific differences in the neuroprotective effect of Simvastatin against alcohol-induced suppression of hippocampal neurogenesis.

#### Distribution of DCX-positive cells

In the female mice (Fig. 3b), the distribution of DCX-positive cells differed significantly across the experimental groups (*p =* 0.000). The distribution of DCX-positive cells in the ALC group was significantly lower than in the control (*p =* 0.000) and SIM (*p =* 0.000) groups, confirming the damaging effects of alcohol on hippocampal neurogenesis. Similarly, the ALC + SIM15 group showed significantly different distribution from both the control (*p =* 0.000) or SIM (*p =* 0.000) groups. Notably, SIM did not significantly differ from the control (*p =* 0.546), suggesting no adverse effects of Simvastatin on hippocampal neurogenesis. A significant difference was observed between the ALC and ALC + SIM5 groups (*p =* 0.022), suggesting that 5 mg Simvastatin partially counteracted alcohol-induced effects. In contrast, no significant difference was found between ALC and ALC + SIM15 (*p =* 0.944), indicating that the higher dose provided limited protection. ALC + SIM5 differed significantly from SIM (*p =* 0.015) or ALC + SIM15 (*p =* 0.034), suggesting a dose-dependent variation in Simvastatin’s efficacy.

A similar trend was observed in the male mice (Fig. 3b), where the distribution of DCX-positive cells in the ALC group was significantly lower than in the control (*p =* 0.001), SIM (*p =* 0.000), or ALC + SIM5 (*p =* 0.000) groups, further confirming the damaging effects of alcohol on hippocampal neurogenesis. However, the distribution of DCX-positive cells was similar between the ALC and ALC + SIM15 groups (*p =* 0.358). Additionally, the ALC + SIM15 group had a lower distribution when compared to the control (*p =* 0.026), SIM (*p =* 0.003), or ALC + SIM5 (*p =* 0.004) groups. In both sexes, the lower dose of Simvastatin reduced alcohol-induced damage, while the higher dose did not, which contrasts with the findings observed for the distribution of PcNA-positive cells, especially in female mice.

## Discussion

Chronic alcohol consumption is well-documented to cause widespread structural and functional brain impairments (Oscar-Berman and Marinkovic 2003; Zahr et al. 2011; Nunes et al. 2019; Rao and Topiwala 2020). Alcohol is a potent amnestic agent (White and Swartzwelder 2004) that particularly disrupts hippocampal function, thereby significantly impairing memory consolidation and retrieval processes (Aggleton 2014; Jauhar et al. 2014; Aggleton and Morris 2018). These disruptions frequently manifest as memory deficits, which are hallmark features of alcohol-related brain damage, especially during critical developmental windows such as adolescence (Hanson et al. 2011; Jacobus and Tapert 2013). In addition to its effects, alcohol inhibits the proliferation and survival of neural progenitor cells in the hippocampus (Nixon and Crews 2002), thereby impairing hippocampal neurogenesis and promoting neurodegeneration (He et al. 2005; Zeigler et al. 2005; Crews et al. 2006; Mira et al. 2020; Doremus-Fitzwater and Deak 2021; Anand et al. 2023). These neurobiological changes underlie many of the cognitive impairments associated with alcohol exposure. Furthermore, neuroimaging studies have revealed that adolescents with a history of alcohol use exhibit significantly reduced hippocampal and prefrontal cortex volumes compared to non-drinking peers (De Bellis et al. 2000; Bellis et al. 2005; Nagel et al. 2005; Oscar-Berman and Marinković 2007), highlighting the vulnerability of the developing brain to alcohol-induced damage.

Alcohol-induced neurodegeneration is closely linked to the upregulation of oxidative stress, a condition characterised by an excessive accumulation of reactive oxygen species (ROS) within cells. These highly reactive molecules cause significant damage to critical biomolecules such as lipids, proteins, and nucleic acids, ultimately impairing cellular integrity and function (Betteridge 2000). Oxidative stress has been implicated in the pathogenesis of various neurodegenerative diseases, including epilepsy, Huntington’s disease, and Parkinson’s disease (Aguiar et al. 2012; Kim et al. 2015). Among brain regions, the hippocampus is particularly vulnerable to alcohol-induced oxidative damage (Enache et al. 2008; Fowler et al. 2014; Rajput et al. 2017; Tsermpini et al. 2022). Alcohol exposure triggers a cascade of detrimental events within the hippocampus, including mitochondrial dysfunction, impaired neuronal signalling, neuronal apoptosis, and suppressed neurogenesis (Hovatta et al. 2010; Brocardo et al. 2011). Compounding these effects, alcohol reduces the activity of key endogenous antioxidant enzymes, such as SOD and GSH-Px, which are essential for cellular detoxification and the maintenance of redox homeostasis (Huang et al. 2009; Contreras-Zentella et al. 2022; Shen et al. 2022). Furthermore, excessive oxidative stress plays a pivotal role in the initiation of apoptosis following exposure to xenobiotic agents such as alcohol (Bhattacharyya et al. 2014; Jelinek et al. 2021), thereby exacerbating neuronal loss and contributing to long-term cognitive and structural deficits.

In the present study, MDA levels across the experimental groups were similar in both sexes. These findings contradict studies that consistently show that alcohol exposure leads to significant increases in MDA levels in the hippocampus of rodents, which is indicative of lipid peroxidation and oxidative stress. Smith et al. (2005), Rajput et al. (2017) and Pamplona-Santos et al. (2019) all reported similar findings, emphasising that chronic alcohol consumption causes oxidative damage in the hippocampus. Furthermore, Zeigler et al. (2005), Tiwari and Chopra (2013) and Pant et al. (2017) highlight that alcohol-induced oxidative stress in the hippocampus can lead to neuronal injury, which may contribute to cognitive deficits. Simvastatin also significantly reduces MDA levels in the hippocampus of rodents, indicating its potential as an antioxidant agent. Eger et al. 2016a, b; Zhang et al. (2016); Jafari et al. (2021) also showed that Simvastatin mitigates oxidative stress in the hippocampus, reducing lipid peroxidation as reflected by lower MDA levels. Cimino et al. (2005) and Lietzau et al. (2023) also reported the neuroprotective benefits of Simvastatin in models of neurodegeneration and ischemia.

Alcohol has been shown to impair hippocampal antioxidant defences, primarily through the reduction of glutathione peroxidase (GSH-Px) activity, therefore promoting oxidative stress and increasing the risk of neurotoxicity (Herrera et al. 2003; Almansa et al. 2013; Tsermpini et al. 2022). Interestingly, conflicting findings have emerged from clinical studies. Wu et al. (2020) reported elevated serum GSH-Px levels in individuals with alcohol use disorder (AUD) compared to healthy controls, a trend similarly observed by Guemouri et al. (1993) and Chen et al. (2011). These discrepancies may reflect compensatory upregulation in peripheral tissues or differences in the chronicity and severity of alcohol exposure, highlighting the complexity of systemic antioxidant responses. In contrast, Simvastatin has been shown to upregulate GSH-Px activity and alleviate oxidative stress (Eger et al. 2016a, b; Zinellu and Mangoni 2021; Mansouri et al. 2022). Its antioxidant and anti-inflammatory properties have been documented across several pathological models, including traumatic brain injury (Lim et al. 2017), senile dementia (Liu et al. 2018), sepsis (Catalão et al. 2017), and alcohol-induced neurotoxicity (Jafari et al. 2021). In these contexts, Simvastatin reduced oxidative damage and preserved neuronal integrity, reinforcing its potential as a neuroprotective agent capable of safeguarding hippocampal function, which is critical for cognitive processes such as learning and memory.

In the present study, GSH-Px activity was highest in the alcohol group in both sexes. A possible explanation for these observations is a compensatory up-regulation of antioxidant enzymes in response to alcohol-induced oxidative stress. However, the elevated alcohol-induced oxidative stress was mitigated by Simvastatin, evident by a significant reduction in GSH-Px activity in the SIM, ALC + SIM5, and ALC + SIM15 groups compared to the alcohol group, indicating its ability to counteract oxidative stress. These findings suggest that Simvastatin mitigated alcohol-induced oxidative stress, as evidenced by a reduction in GSH-Px activity, thereby reinforcing its antioxidant capacity even under conditions of elevated ROS production. In support of the present findings, ROS are known to upregulate antioxidant enzymes such as GSH-Px as part of the cellular adaptive defence mechanism. This response is primarily mediated by redox-sensitive transcription factors like Nrf2, which enhance the expression of antioxidant genes in response to oxidative stress (Ma 2013; Pizzino et al. 2017; Zhang et al. 2017a, b). Conversely, under conditions of excessive or prolonged oxidative stress, ROS can cause oxidative damage to these enzymes, thereby impairing their function (Pizzino et al. 2017).

SOD is another key antioxidant enzyme that helps maintain redox balance and detoxifies harmful molecules in living organisms. It plays a crucial role as the first line of defence against reactive nitrogen species (RNS), ROS, and other dangerous molecules by catalysing the conversion of O_2_^−^ into molecular oxygen (O_2_) and H_2_O_2_ (Younus 2018; Chidambaram et al. 2024). Studies show that chronic alcohol use leads to a decrease in SOD activity, disrupting the brain’s antioxidant defence system which may contribute to neurological dysfunction and cognitive deficits (Marklund et al. 1983; Huang et al. 2009; Yang et al. 2022; Kado et al. 2024). On the otherhand, Simvastatin boosts SOD activity thereby increasing its antioxidant effect to counteract oxidative damage in the hippocampus (Eger et al. 2016a, b; Catalão et al. 2017; Zhang et al. 2017a, b; Liu et al. 2018). In an experimental model of sepsis (Catalão et al. 2017) and alcohol-induced neurotoxicity model (Jafari et al. 2021), Simvastatin has been shown to protect the hippocampus from oxidative damage by increasing SOD activity, further confirming its neuroprotective properties. Additionally, in an Alzheimer’s disease model (Adeli et al. 2017), Simvastatin prevented cognitive decline by increasing SOD activity.

In the present study, the effect of chronic alcohol on SOD activity in the mice was found to be non-significant in both sexes, which aligns with some studies that suggest alcohol may not always affect SOD activity in specific tissues or under certain conditions. For instance, Enache et al. (2008) reported no significant changes in SOD activity in rat brains following chronic alcohol exposure in a prenatal restraint stress rat model, suggesting that alcohol-induced oxidative stress may not always result in direct alterations to SOD levels in all tissues. The lack of change in SOD activity may be attributed to several factors, including tissue-specific antioxidant responses, the duration and dosage of alcohol exposure, or adaptive mechanisms that compensate for alcohol-induced oxidative stress.

The differences between the present and previous studies highlight the complex, multifactorial nature of oxidative damage and antioxidant regulation (Singh and Singh 2008). It is possible that other enzymes or mechanisms, such as GSH-Px, were more prominently upregulated to counteract the ROS generated during alcohol metabolism. This may reflect a shift in the antioxidant defence system away from SOD, suggesting that alternative enzymatic pathways could be more responsive to alcohol-induced oxidative stress in rodent models. As highlighted by Ruiter-Lopez et al. (2025), alcohol-induced oxidative stress does not uniformly affect all antioxidant systems in all tissues, which suggests that future studies should consider a more nuanced approach, taking into account the tissue-specific antioxidant responses and mechanisms that may be activated in the presence of alcohol. Furthermore, other factors, such as the strain of rodents used, age, and gender, may influence the antioxidant defence system, complicating the interpretation of results.

Neurogenesis, the process of generating new neurons from neural stem cells, continues from adolescence into adulthood, primarily in the subventricular zone (SVZ) of the lateral ventricles and the SGZ of the DG in the hippocampus (Crews et al. 2006; Gil-Perotín et al. 2009; Lazarov and Hollands 2016). In the SGZ, neural progenitor cells proliferate, differentiate into glial cells or neurons, and migrate into the GCL, where they integrate into hippocampal circuits involved in learning, memory (Shors et al. 2001), and mood regulation (Malberg et al. 2000). Neurogenesis is particularly active during adolescence, occurring at rates up to four times higher than in adulthood, contributing to increased granule cell density and hippocampal volume (Sousa et al. 1998; Hueston et al. 2017; Boldrini et al. 2018). Various intrinsic factors (e.g., neurotrophic factors, neurotransmitters) and extrinsic factors (e.g., stress, alcohol, and environmental stimuli) regulate hippocampal neurogenesis (Åberg et al. 2005; Cameron and Glover 2015; Opendak et al. 2016).

Alcohol exposure, particularly during adolescence, induces neurotoxicity, leading to cognitive impairments and altered hippocampal function (White and Swartzwelder 2004; Zeigler et al. 2005; Peeters et al. 2014; Risher et al. 2015). In this study, neurogenic patterns remained consistent across experimental groups, with no significant morphological changes observed following alcohol exposure or Simvastatin treatment, aligning with previous findings on adolescent hippocampal neurogenesis (Crews et al. 2006; Broadwater et al. 2014; Robin et al. 2014). However, proliferative cell distribution (PcNA- and DCX-positive cells) was significantly reduced in the alcohol-exposed group compared to others, confirming the inhibitory effects of alcohol on neurogenesis.

These findings align with previous studies reporting alcohol-induced neurogenic suppression. Chronic alcohol exposure in adult rats reduced PcNA-positive cells in the DG, indicating disrupted neural progenitor proliferation (He et al. 2005; Morris et al. 2010; Tiwari and Chopra 2013; Broadwater et al. 2014). Similarly, alcohol decreased DCX-positive immature neurons, impairing neuronal differentiation (He et al. 2005; Le Maître et al. 2018; Olateju et al. 2018). Broadwater et al. (2014) also reported fewer DCX-expressing neurons in adolescent rodents after intermittent alcohol exposure. Morris et al. (2010) also found that chronic alcohol exposure significantly decreased both PcNA- and DCX-positive cells in the DG, but not in the SVZ, indicating a region-specific effect of alcohol on neurogenesis. Nixon and Crews (2002) found that acute and binge alcohol exposure significantly suppressed the proliferation of progenitor cells in the DG. Chronic alcohol exposure further decreased proliferating cells in the DG of adult rats (Herrera et al. 2003), highlighting its long-term neurotoxic effects. These rodent findings align with human studies, where individuals with alcohol use disorders exhibit reduced hippocampal neurogenesis (Crews et al. 2004; Taffe et al. 2010).

Alcohol exerts its neurodegenerative effects by significantly inducing oxidative stress, which directly damages the mitochondrial structure, leading to the death of neural progenitor cells (Nixon and Crews 2002). This is also supported by the fact that elevated oxidative stress resulted in a reduction of DCX-positive cells and inhibition of neuronal differentiation (Nixon and Crews 2002; Ye et al. 2015). In the present study, increased GSH-Px activity, indicative of elevated oxidative stress, was observed in the alcohol-treated group compared to the control, alongside a corresponding reduction in the distribution of proliferative cells and immature neurons within the hippocampus of adolescent mice. In addition, alcohol-induced inflammation may inhibit the proliferation of neural progenitor cells, as activation of microglia and astrocytes leads to increased secretion of pro-inflammatory cytokines (e.g., TNF-α, IL-6), which are known to suppress neural progenitor cell proliferation and disrupt the differentiation of DCX-positive neurons (Zonis et al. 2013, 2015; Walter and Crews 2017). Similarly, alcohol-induced suppression of neurotrophic factors like BDNF may reduce neural progenitor cells, providing another mechanism for impaired hippocampal neurogenesis (Tateno et al. 2004; Silva-Peña et al. 2019).

The lower concentration (5 mg) of Simvastatin consistently counteracted the inhibitory effects of alcohol by increasing the distribution of PcNA- and DCX-positive cells in the hippocampus of both female and male adolescent mice. However, the higher concentration (15 mg Simvastatin) was not beneficial, except for the increased distribution of PcNA-positive cells in female mice, which may indicate possible sex-specific differences in the effect of Simvastatin on neurogenesis. These results are consistent with previous reports that Simvastatin enhances neural progenitor cell proliferation in the hippocampus by upregulating PcNA and other cell cycle-related proteins (Lu et al. 2007; Wang et al. 2015; Xie et al. 2015; Trigiani et al. 2019) by increasing the population of DCX-positive cells in the dentate gyrus of stroke-induced rats, further supporting its neuroprotective role (Chen et al. 2016). Additionally, Simvastatin has been shown to increase DCX expression, thereby promoting neuronal differentiation and the maturation of newly generated neurons (Wang et al. 2015; Xie et al. 2015). Simvastatin’s ability to attenuate alcohol-induced suppression of neurogenesis is thought to be mediated by its anti-inflammatory effects (Zhou et al. 2017), upregulation of neurotrophic factors (Wu et al. 2008) and enhancement of synaptic plasticity (Chen et al. 2016).

In conclusion, this study provides evidence that Simvastatin mitigates the adverse effects of adolescent alcohol exposure on hippocampal neurogenesis. Alcohol significantly impaired cell proliferation and immature neuron formation in the hippocampus, which are processes critical for cognitive function, memory, and learning. Simvastatin modulated antioxidant activity and enhanced neurogenesis in a sex- and dose-dependent manner, with 15 mg being more effective in females and 5 mg in males. However, a key limitation is the absence of behavioural assessments to confirm functional cognitive outcomes. While Simvastatin shows promise as a neuroprotective agent, its long-term effects on brain function warrant caution. Cholesterol plays a vital role in myelination and neural integrity, and prolonged Simvastatin use may disrupt these processes, potentially leading to cognitive side effects (see review by Schultz et al. 2018). Future studies should incorporate cognitive testing and explore alternative protocols, including preventive (pre-treatment) and reversal (post-alcohol exposure) models, to better assess the therapeutic potential of Simvastatin. Overall, these findings highlight Simvastatin’s potential role in mitigating alcohol-induced hippocampal damage during adolescence.

## Data Availability

The datasets generated and/or analyzed during the current study emanated from the PhD study of RdP. They are raw values obtained from oxidative stress assays and the distribution of proliferative cells. They will be made available upon request.
